# Trends and Outcomes of Aortic Valve Replacement in Patients With Diabetes in the US

**DOI:** 10.3389/fcvm.2022.844068

**Published:** 2022-03-18

**Authors:** Sarah Khan, Soha Dargham, Jassim Al Suwaidi, Hani Jneid, Charbel Abi Khalil

**Affiliations:** ^1^Research Department, Weill Cornell Medicine-Qatar, Doha, Qatar; ^2^Heart Hospital, Hamad Medical Corporation, Doha, Qatar; ^3^The Michael E. DeBakey VA Medical Centre, Baylor College of Medicine, Houston, TX, United States; ^4^Joan and Sanford I. Weill Department of Medicine, Weill Cornell Medicine, New York, NY, United States

**Keywords:** aortic stenosis, aortic valve repair, SAVR, TAVR, diabetes

## Abstract

**Aims:**

We aimed to assess the trend and outcome of aortic valve replacement in patients with diabetes.

**Background:**

Diabetes is associated with higher cardiovascular events.

**Methods:**

Data from the National Inpatient Sample was analyzed between 2012 and 2017. We compared hospitalizations and in-hospital cardiovascular outcomes in patients with diabetes to those without diabetes, hospitalized for aortic valve replacement.

**Results:**

In diabetes patients undergoing TAVR, the mean age of participants decreased from 79.6 ± 8 to 67.8 ± 8, hospitalizations increased from 0.97 to 7.68/100,000 US adults (*p* < 0.002 for both). There was a significant temporal decrease in mortality, acute renal failure (ARF), and stroke. Compared to non-diabetic patients, those with diabetes had a higher risk of stroke, ARF, and pacemaker requirement [adjusted OR = 1.174 (1.03–1.34), 1.294 (1.24–1.35), 1.153 (1.11–1.20), respectively], but a similar adjusted mortality risk. In diabetes patients undergoing sAVR, the mean age of participants decreased from 70.4 ± 10 to 68 ± 9 (*p* < 0.001), hospitalizations dropped from 7.72 to 6.63/100,000 US adults (*p* = 0.025), so did mortality, bleeding, and ARF. When compared to non-diabetes patients, those with diabetes were older and had a higher adjusted risk of mortality, stroke, and ARF [adjusted OR= 1.115 (1.06–1.17), 1.140 (1.05–1.23), 1.217 (1.18–1.26); respectively].

**Conclusion:**

The recent temporal trend of aortic valve replacement in patients with diabetes shows a significant increase in TAVR coupled with a decrease in sAVR. Mortality and other cardiovascular outcomes decreased in both techniques. sAVR, but not TAVR, was associated with higher in-hospital mortality risk.

## Introduction

Aortic stenosis (AS) is characterized by progressive aortic valve dysfunction. It affects 0.2% of the asymptomatic American population between 50 and 59 years and 8.9% of people by age 80; despite its relatively low prevalence, AS is burdensome on healthcare due to its progressive nature and high mortality when it becomes symptomatic. Though mortality is not increased in asymptomatic patients, it rises to 50% when they become symptomatic (1). There also does not currently exist any medical therapy to prevent or impede the progression of AS severity; thus, the mainstay of management for AS is replacement therapy once it becomes symptomatic.

Diabetes is associated with an increased macrovascular disease risk (2). It is also a significant risk factor for the development of aortic stenosis. Diabetes causes left ventricular remodeling and dysfunction, further compounding the changes brought on by aortic stenosis. Diabetes has also worsened ventricular remodeling caused by aortic stenosis due to altered myocyte structure and fibrosis (3).

Few clinical studies have attempted to elucidate the relationship between diabetes and aortic valve replacement (4–8). Further, results from these studies were conflicting and mainly limited to surgical aortic valve replacement. Our study examines the burden of diabetes mellitus on cardiovascular and economic outcomes in patients undergoing aortic valve replacement either by transcatheter aortic valve replacement (TAVR) or surgical aortic valve replacement (sAVR).

## Methods

### Data Extraction

Our study was conducted using data from the National Inpatient Sample (NIS) database, the largest all-payer database in the US. Developed as a part of the Healthcare Cost and Utilization Project (HCUP) by the Agency for Healthcare Research and Quality (AHRQ), the NIS serves as a federal-state joint initiative to provide national and state levels for encounter-level research. Discharge information for encounters includes patient characteristics, clinical outcomes, and economic data. The NIS draws data from all states participating in HCUP. It has data from roughly 7 million hospital stays annually, accounting for 20% of yearly national discharges and about 95% after weighting. Data weighting was used to allow for representative nationwide population estimates as recommended by the Healthcare Cost and Utilization Project, to which the NIS belongs (9). The database promotes patient confidentiality by providing de-identified data that may be extracted through the International Classification of Diseases (ICD) codes. The study received administrative IRB approval as it contains only de-identified data (record number 18-00017).

### Diagnosis and Outcomes

The NIS was queried for all patients undergoing aortic valve replacement, either transcatheter aortic valve implantation (TAVI) or surgical aortic valve replacement (sAVR), between 2012 and 2017. Further, patients were classified into diabetics and non-diabetics. Diabetes was only limited to type 2 diabetes. Data were extracted using ICD-9 and ICD-10 codes, validated in aortic valve replacement and diabetes studies from the NIS database (4, 10–12). Patients under 18 years of age or with missing data for age, gender, in-hospital mortality, or hospital charges were excluded from the analysis. The primary outcome was mortality. Secondary cardiovascular outcomes included: post-procedural stroke, bleeding, vascular complications, acute renal failure, and pacemaker requirement. Secondary socio-economic outcomes were the length of stay (LoS) and total charges per stay.

### Statistical Analysis

Baseline patient clinical characteristics are presented as means (± standard deviation), medians (interquartile range), or numbers (percentage) as appropriate. Patient-level discharge trend weights consisted of applying the TRENDWT variable.

Comparison of variables between diabetes and non-diabetes patients in every aortic valve replacement group was made using the *t*-test for continuous variables, χ^2^ test for categorical tests. All continuous variables were assessed for normality graphically and statistically using Shapiro Wilk's test. Temporal trend in every group was assessed using a trend test.

In-hospital mortality was adjusted for age, then stratified by sex. Data on hospitalizations was presented as the number of hospitalizations per 100,000 of the US population. To account for inflation, costs were corrected using rates provided by the US Bureau of labor statistics.

The patient's comorbidity burden was represented through the Elixhauser score, which includes 31 baseline characteristics associated with a worse outcome, as previously described (13). Unadjusted odds ratios were calculated to examine differences in the outcomes between diabetics and non-diabetics undergoing TAVR and sAVR. Adjusted odds ratios were then calculated through multivariate regression analysis, including baseline variables that were significantly different between both groups. Multivariate models were also constructed to identify predictors of mortality among diabetic patients undergoing TAVR and sAVR, respectively. All statistical analyses were performed using SPSS (IBM, version 26.0, NY) with a *p* < 0.05 considered to represent statistical significance.

## Results

### Population Analyzed

**Figure 1:**
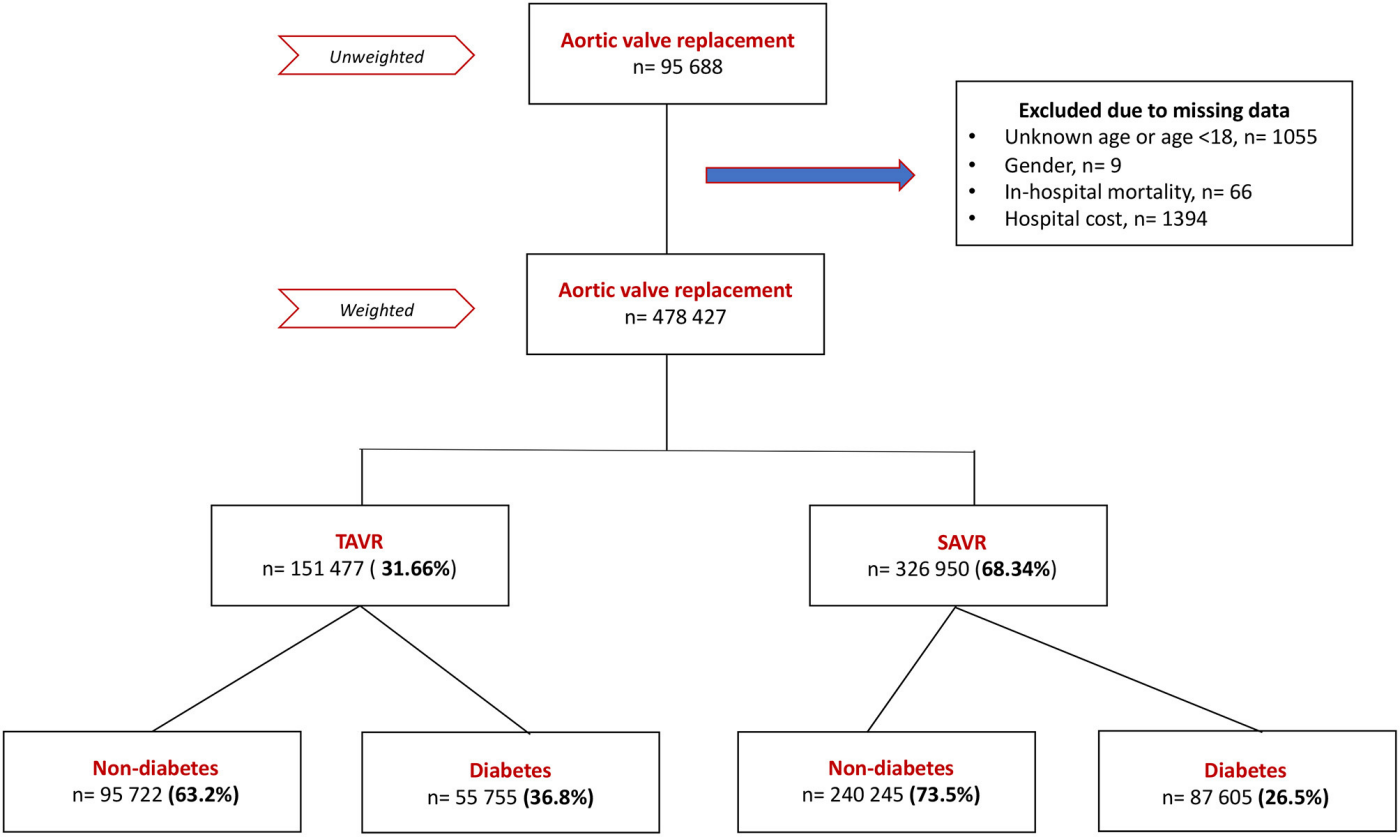
Flow chart of the study.

A total of 428,427 patients undergoing aortic valve replacement (AVR) were included in the cohort after excluding patients with missing data and weighting (Figure 1); 31.7% of them were hospitalized for TAVR and 68.3% for sAVR. The diabetes population constituted 36.8% of TAVR patients and 26.5% of sAVR patients.

### Patients Undergoing TAVR

#### Trend

**Figure 2:**
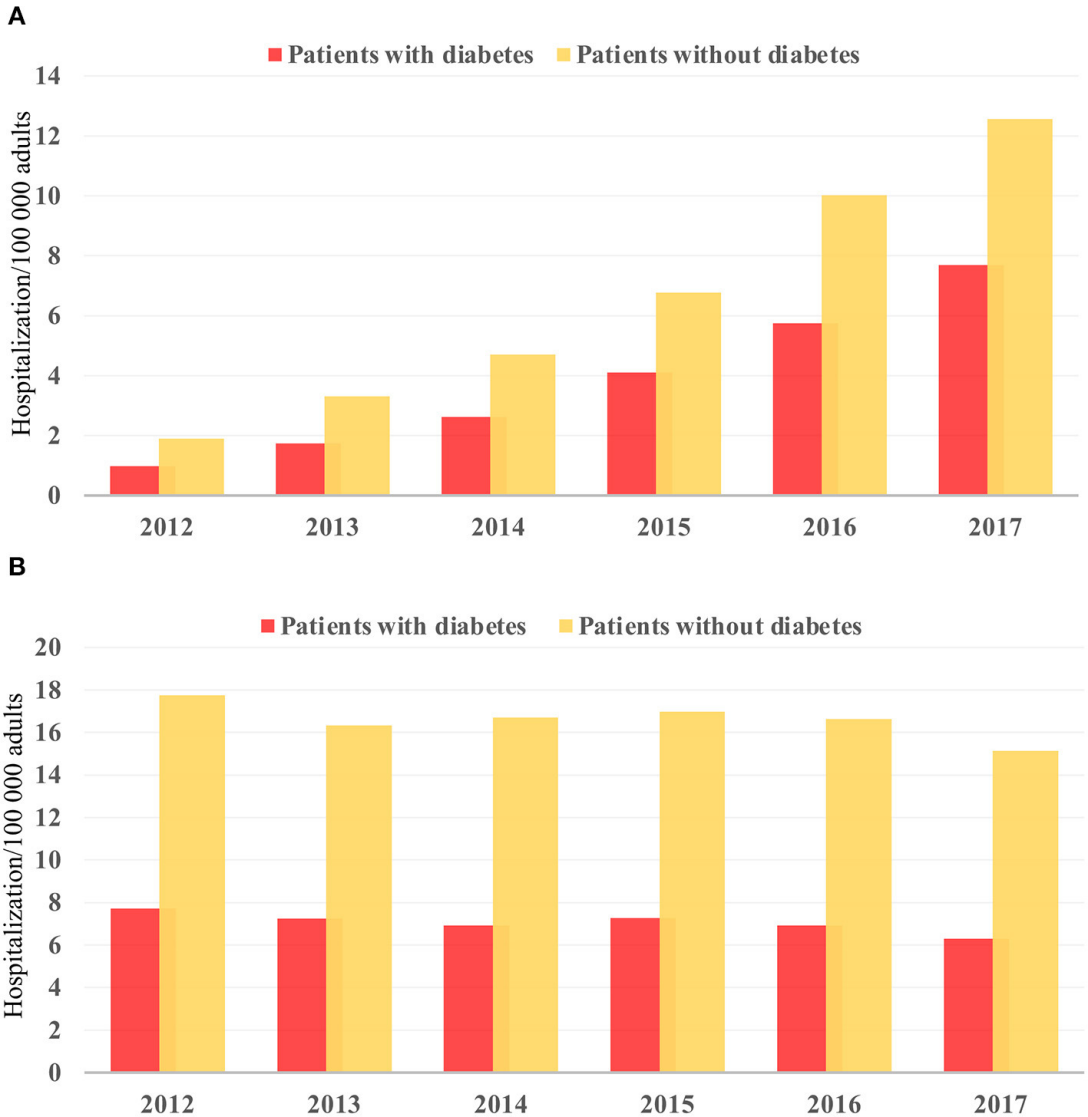
Temporal trend in hospitalization/100,000 US adults from 2012 to 2017 in **(A)** TAVR and **(B)** sAVR patients.

**Figure 3:**
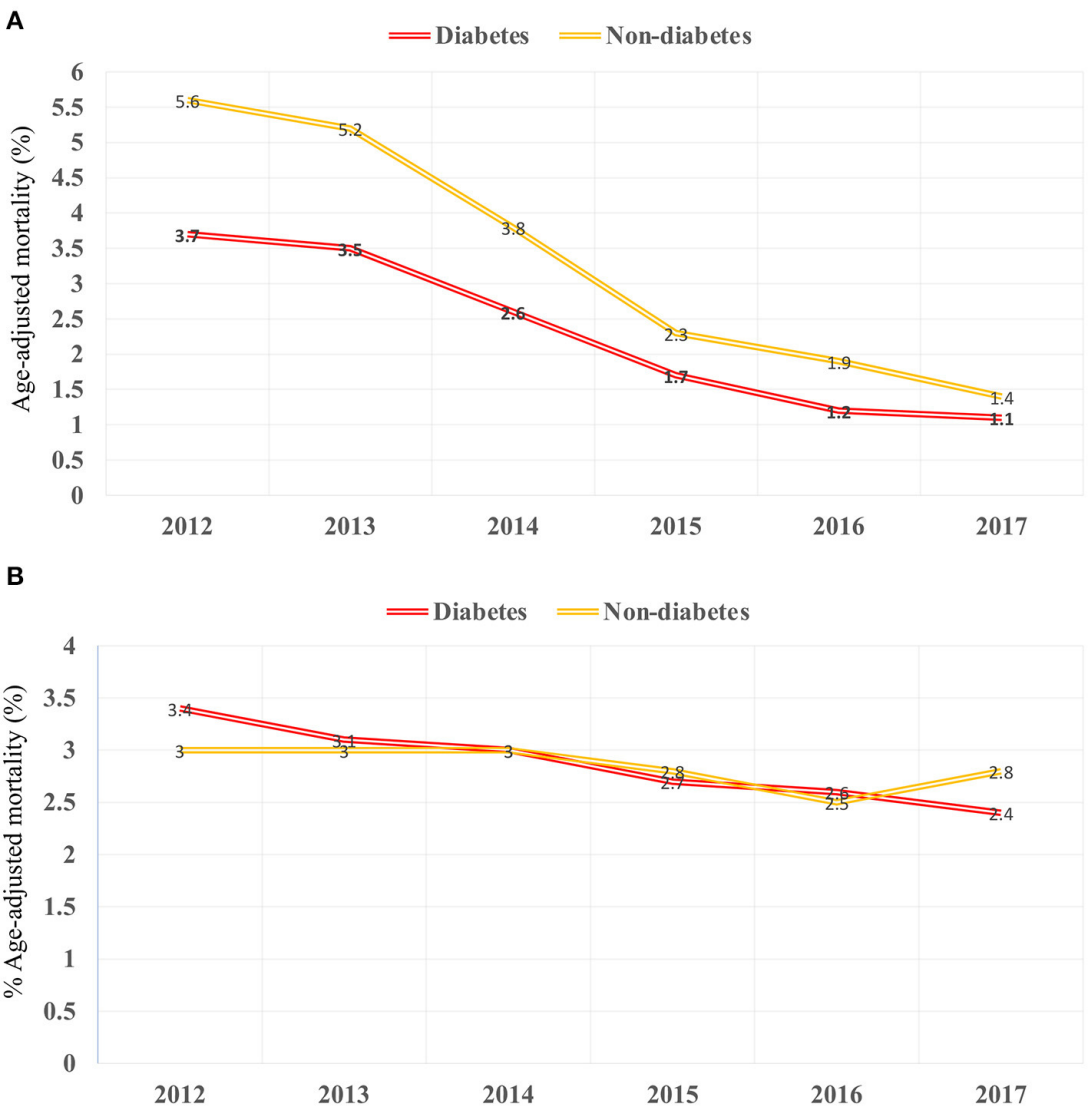
Temporal trend in mortality from 2012 to 2017 in **(A)** TAVR and **(B)** sAVR patients.

**Figure 4:**
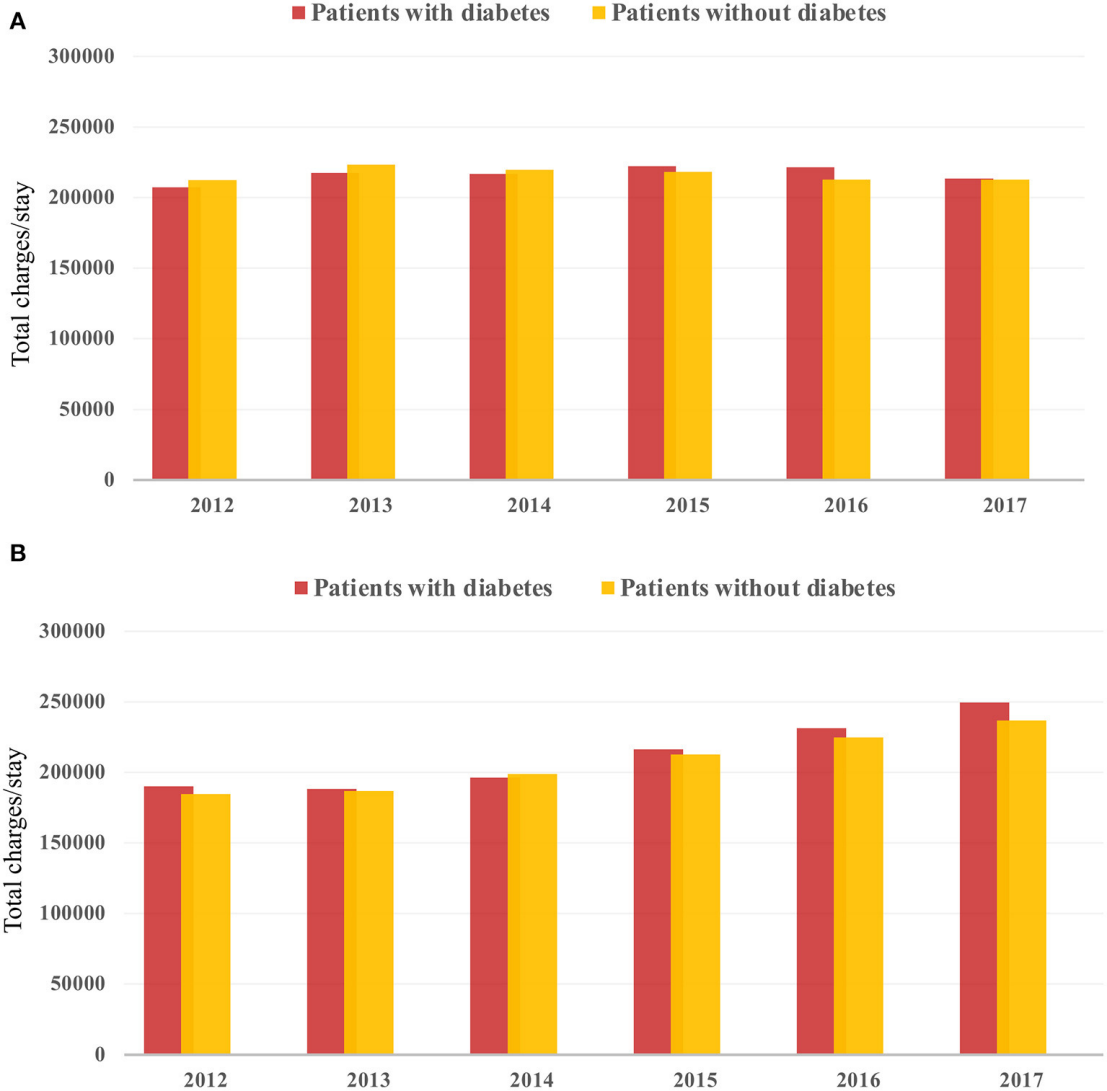
Temporal trend in total hospital charges from 2012 to 2017 in **(A)** TAVR and **(B)** sAVR patients.

There was an increase in hospitalization from 0.97 to 7.68/100,000 US adults from 2012 to 2017 (*p* < 0.001; Figure 2A) in diabetes patients hospitalized for TAVR. Mean age decreased from 79.69 to 77.89 (*p* < 0.002; Table 1), while remaining stable in non-diabetic patients (Supplementary Table 1). The proportion of patients undergoing TAVR who were >85 years of age decreased, while those aged 55–64 and 65–74 years increased (*p* < 0.05 for all). Slightly more men underwent TAVR than women, and this proportion rose linearly. Patient demographic analysis revealed a marginal rise in the proportion of Hispanic patients undergoing the procedure (*p* = 0.029). There was an increasing trend in patients undergoing TAVR with obesity (*p* = 0.016) and smoking (*p* < 0.001), while a negative trend was observed in patients with peripheral vascular disease, the prevalence of which decreased from 32.5 to 19.8% (*p* = 0.003). Age-adjusted mortality in patients with diabetes decreased significantly from 3.7% in 2012 to 1.1% in 2017 (*p* = 0.001; Figure 3A). A similar trend was observed in patients without diabetes whose mortality decreased from 5.6 to 1.4%. Of the in-hospital complications studied, stroke rate decreased from 1.5 to 0.5% (*p* = 0.019) and acute renal failure rate decreased from 18.2 to 12.3% (*p* = 0.033), which was also observed in their non-diabetic counterparts (Supplementary Table 1). There was no trend in the proportion of patients who suffered post-TAVR bleeding or pacemaker requirement. Length of hospital stay among these patients also reduced from 8(6) days in 2012 to 4(4) days in 2017 (*p* < 0.001). There was no trend, however, in total charges/stay (Figure 4A).

**Table 1:** Baseline characteristic of patients with diabetes undergoing TAVR.

	**2012**	**2013**	**2014**	**2015**	**2016**	**2017**	***P*** **-value**
**Age**							
Mean (SD)	79.69 (8.231)	79.10 (8.295)	79.01 (8.090)	78.95 (8.201)	78.41 (8.167)	77.89 (8.023)	0.002
<55	25 (1.1)	55 (1.3)	50 (0.8)	95 (0.9)	115 (0.8)	135 (0.7)	0.052
55–64	85 (3.7)	170 (4.1)	310 (4.9)	490 (4.8)	655 (4.7)	985 (5.2)	0.02
65–74	460 (20.2)	755 (18.4)	1,305 (20.8)	2,115 (20.9)	3,470 (24.7)	4,730 (25.0)	0.022
75–84	950 (41.7)	1,980 (48.2)	2,740 (43.7)	4,495 (44.5)	6,030 (42.8)	8,930 (47.1)	0.638
>84	760 (33.3)	1,150 (28.0)	1,860 (29.7)	2,910 (28.8)	3,805 (27.0)	4,160 (22.0)	0.023
**Gender**							
Male	1,230 (53.9)	2,240 (54.5)	3,420 (54.6)	5,585 (55.3)	7,950 (56.5)	10,895 (57.7)	0.002
Female	1,050 (46.1)	1,870 (45.5)	2,845 (45.4)	4,520 (54.7)	6,125 (43.5)	8,045 (42.3)	0.002
**Race**							
White	1,745 (82.7)	3,265 (86.4)	5,050 (86.7)	8,115 (86.0)	11,035 (83.0)	15,365 (84.4)	0.891
Black	80 (3.8)	170 (4.5)	350 (6.0)	420 (4.4)	790 (5.9)	930 (5.1)	0.083
Hispanic	90 (4.3)	125 (3.3)	250 (4.3)	460 (4.9)	795 (6.0)	1,075 (5.9)	0.029
Asian	30 (1.4)	65 (1.7)	55 (0.9)	145 (1.5)	195 (1.5)	330 (1.8)	0.509
Native American	25 (1.2)	10 (0.3)	5 (0.1)	15 (0.2)	25 (0.2)	80 (0.4)	0.254
Other	140 (6.6)	145 (3.8)	115 (2.0)	285 (3.0)	460 (3.5)	415 (2.3)	0.127
**Income**							
Low	565 (25.1)	920 (22.8)	1,510 (24.7)	2,395 (24.7)	3,415 (24.7)	4,275 (22.9)	0.594
Low-Mid	530 (23.6)	1,110 (27.0)	1,655 (27.0)	2,555 (25.6)	3,635 (26.3)	5,160 (27.7)	0.187
High-Mid	640 (28.4)	1,000 (24.8)	1,510 (24.7)	2,665 (26.7)	3,505 (25.3)	4,695 (25.2)	0.354
High	515 (22.9)	1,010 (25.0)	1,450 (23.7)	2,385 (23.8)	3,290 (23.8)	4,530 (24.3)	0.607
**Primary expected payer**							
Medicare	2,050 (89.9)	3,625 (88.6)	5,620 (89.8)	9,030 (89.4)	12,600 (89.6)	16,805 (88.8)	0.658
Medicaid	45 (2.0)	20 (0.5)	80 (1.3)	115 (1.1)	225 (1.6)	265 (1.4)	0.984
Private insurance	145 (6.4)	300 (7.3)	455 (7.3)	725 (7.2)	945 (6.7)	1,390 (7.3)	0.468
Self pay	10 (0.4)	25 (0.6)	15 (0.2)	50 (0.5)	90 (0.6)	75 (0.4)	0.015
No charge	0 (0.0)	10 (0.2)	0 (0.0)	5 (0.0)	0 (0.0)	0 (0.0)	0.441
Other	30 (1.3)	110 (2.7)	85 (1.4)	180 (1.8)	195 (1.4)	380 (2.0)	1
**Comorbidities**							
Obesity	540 (23.7)	925 (22.5)	1,450 (23.1)	2,550 (25.2)	3,770 (26.8)	5,455 (28.8)	0.016
Hypertension	1,935 (84.9)	3,525 (85.5)	5,445 (86.9)	9,005 (89.1)	12,020 (85.4)	12,020 (85.4)	0.226
Smoking	540 (23.7)	1,125 (27.4)	1,885 (30.1)	3,275 (32.4)	4,780 (34.0)	6,870 (36.3)	<0.001
Dyslipidemia	1,520 (66.7)	2,995 (72.9)	4,670 (74.5)	7,405 (73.3)	10,325 (73.4)	13,860 (73.2)	0.186
Elixhauser score, *n* (SD)	7.7494 (9.58948)	7.8356 (9.85215)	9.3032 (10.02094)	7.2013 (9.21887)	9.6867 (8.21952)	9.0122 (7.52814)	0.006
**Past medical history**							
Peripheral vascular disease	740 (32.5)	1,295 (31.5)	1,805 (28.8)	2,835 (28.1)	3,550 (25.2)	3,745 (19.8)	0.003
Renal failure	915 (40.1)	1,740 (42.3)	2,605 (41.6)	4,380 (43.3)	6,035 (42.9)	7,830 (41.3)	0.388
Coronary artery disease	1,660 (72.8)	3,115 (75.8)	4,555 (72.7)	7,650 (75.57)	10,645 (75.6)	14,080 (74.3)	0.476
**Hospital bed size**							
Small	17 (3.7)	17 (2.1)	68 (5.4)	104 (5.1)	159 (5.6)	233 (6.2)	0.055
Medium	57 (12.5)	125 (15.2)	221 (17.6)	426 (21.1)	511 (18.2)	732 (19.3)	0.049
Large	382 (83.8)	680 (82.7)	964 (76.9)	1,491 (73.8)	2,145 (76.2)	2,823 (74.5)	0.024
**Hospital location**							
Rural	5 (1.1)	8 (1.0)	7 (0.6)	12 (0.6)	25 (0.9)	38 (1.0)	0.707
Urban	451 (98.9)	814 (99.0)	1,246 (99.4)	2,009 (99.4)	2,790 (99.1)	3,750 (99.0)	0.707
**Hospital region**							
Northeast	108 (23.7)	226 (27.5)	359 (28.7)	482 (23.8)	613 (21.8)	860 (22.7)	0.287
Midwest	585 (25.7)	955 (23.2)	1,450 (23.1)	2,335 (23.1)	3,480 (24.7)	4,540 (24.0)	0.702
South	900 (39.5)	1,665 (40.5)	2,320 (37.0)	3,415 (33.8)	4,885 (34.7)	6,525 (34.5)	0.026
West	255 (11.2)	360 (8.8)	700 (11.2)	1,945 (19.2)	2,645 (18.8)	3,575 (18.9)	0.029
**Outcomes**							
Stroke	35 (1.5)	80 (1.9)	60 (1.0)	85 (0.8)	95 (0.7)	95 (0.5)	0.019
Pacemaker	210 (9.2)	380 (9.2)	750 (12.0)	1,140 (11.3)	1,720 (12.2)	2,050 (10.8)	0.156
Bleeding	65 (2.9)	160 (3.9)	235 (3.8)	370 (3.7)	530 (3.8)	650 (3.4)	0.566
Acute renal failure	415 (18.2)	895 (21.8)	1,180 (18.8)	1,740 (17.2)	2,030 (14.4)	2,335 (12.3)	0.033
Length of stay	8 (5)	8 (7)	7 (6)	6 (6)	5 (5)	4 (4)	<0.001

#### Comparison of Diabetes to Non-diabetes Patients

In a comparison of baseline demographic variables between diabetes and non-diabetes, mean age was found to be younger among diabetic patients (78.51 ± 8 years) relative to non-diabetic patients (81.49 ± 8 years) (*p* < 0.001; Table 2). As expected, obesity, hypertension, dyslipidemia, smoking, renal failure, coronary artery disease were more likely to be prevalent in diabetes (*p* < 0.001 for all). Interestingly, PVD was more prevalent in non-diabetes patients (*p* < 0.001). Unadjusted analysis initially showed lower mortality among diabetic patients compared to non-diabetics [OR = 0.674 (0.63–0.73), *p* < 0.001]. However, on adjusted analysis, this finding was no longer significant [OR = 0.929 (0.85–1.01), *p* = 0.09; Table 3]. Following adjustment, the following complications occurred more frequently among diabetics: stroke [OR=1.174 (1.03–1.34), *p* = 0.014], pacemaker requirement [OR = 1.153 (1.11–1.20), *p* < 0.001], and acute renal failure [1.294 (1.24–1.35), *p* < 0.001]. Conversely, bleeding was less likely [OR = 0.936 (0.88–0.99), *p* = 0.033]. Unsurprisingly, patients with diabetes had a slightly longer mean LoS [6(6) vs. 5(4) days, diabetes vs. non-diabetes, *p* < 0.01], and total charges [182.242 (135,339–258,400) vs. 180,235 (133,686–255,793) USD, diabetes vs. non-diabetes, *p* < 0.01].

**Table 2:** Comparison of patients with vs. without diabetes undergoing TAVR.

		**Diabetes**	**Non-diabetes**	***P*** **-value**
	Mean (sd)	78.51 (8.146)	81.49 (8.347)	<0.001
Age	<55	475 (0.9%)	1,200 (1.3%)	<0.001
	55–64	2,695 (4.8%)	3,045 (3.2%)	<0.001
	65–74	12,835 (23.0%)	11,960 (12.5%)	<0.001
	75–84	25,125 (45.0%)	36,930 (38.6%)	<0.001
	>84	14,645 (26.3%)	42,580 (44.5%)	0.021
Gender	Male	31,320 (56.2%)	49,385 (51.6%)	<0.001
	Female	24,455 (43.8%)	46,330 (48.4%)	<0.001
Race	White	44,575 (84.7%)	80,785 (89.3%)	<0.001
	Black	2,740 (5.2%)	3,110 (3.4%)	<0.001
	Hispanic	2,795 (5.3%)	3,115 (3.4%)	<0.001
	Asian	820 (1.6%)	855 (0.9%)	<0.001
	Native American	160 (0.3%)	185 (0.2%)	<0.001
	Other	1,560 (0.3%)	2,440 (2.7%)	<0.001
Income	Low	13,080 (23.8%)	19,045 (20.2%)	<0.001
	Low-Mid	14,645 (26.7%)	23,515 (25.0%)	<0.001
	High-Mid	14,015 (25.5%)	25,275 (26.8%)	<0.001
	High	13,180 (24.0%)	26,335 (28.0%)	<0.001
Primary expected payer	Medicare	49,730 (89.3%)	86,795 (90.8%)	<0.001
	Medicaid	750 (1.3%)	915 (1.0%)	<0.001
	Private insurance	3,960 (7.1%)	6,320 (6.6%)	<0.001
	Self pay	265 (0.5%)	445 (0.5%)	0.62
	No charge	15 (0.0%)	25 (0.0%)	0.888
	Other	980 (1.8%)	1,060 (1.1%)	<0.001
Obesity		14,690 (26.3%)	11,090 (11.6%)	<0.001
Hypertension		43,725 (78.4%)	67,550 (70.6%)	<0.001
Smoking		18,475 (33.1%)	30,665 (32.0%)	<0.001
Dyslipidemia		40,775 (73.1%)	60,360 (63.1%)	<0.001
Peripheral vascular disease		13,970 (25.0%)	25,710 (26.9%)	<0.001
Renal Failure		41,705 (74.8%)	65,185 (68.1%)	<0.001
Coronary artery disease		41,705 (74.8%)	65,185 (68.1%)	<0.001
Elixhauser score, mean (SD)		10.0233 (8.835)	8.8019 (8.619)	<0.001
Hospital bedsize	Medium	10,360 (18.6%)	17,550 (18.3%)	0.068
	Large	42,425 (76.1%)	72,780 (76.0%)	0.18
	Rural	475 (0.9%)	850 (0.9%)	0.039
Hospital location	Urban	55,300 (99.1%)	94,865 (99.1%)	0.463
	Northeast	13,240 (23.7%)	24,870 (26.0%)	<0.001
Hospital Region	Midwest	13,345 (23.9%)	21,575 (22.5%)	<0.001
	South	19,710 (35.5%)	32,585 (34.0%)	<0.001
	West	9,480 (17.0%)	16,685 (17.4%)	<0.001

**Table 3:** Comparison of outcomes of patients undergoing SAVR and TAVR, with and without diabetes.

	**TAVR**				**sAVR**			
	**Number of events** **(OR; 95% CI)**		**Adjusted OR** **(95% CI)**	***P*** **-value**	**Number of events** **(OR; 95% CI)**		**Adjusted OR** **(95% CI)**	***P*** **-value**
**Outcomes**	**No diabetes**	**Diabetes**			**No diabetes**	**Diabetes**	
Mortality	2,360 (OR = 1)	935 (OR = 0.674; 0.63-0.73)	0.929 (0.85–1.01)	0.09	6,795 (OR = 1)	2,950 (OR = 1.021; 0.98–1.07)	1.115 (1.06–1.17)	<0.001
Stroke	875 (OR = 1)	450 (OR = 0.882; 0.77–0.99)	1.174 (1.03–1.34)	0.014	2,710 (OR = 1)	1,065 (OR = 0.923; 0.86–0.99)	1.140 (1.05–1.23)	0.001
Pacemaker requirement	9,650 (OR = 1)	6,250 (OR = 1.126; 1.09–1.16)	1.153 (1.11–1.20)	<0.001	12,840 (OR = 1)	5,800 (OR = 1.065; 1.03–1.10)	1.089 (1.05–1.13)	<0.001
Bleeding	4,090 (OR = 1)	2,010 (OR = 0.838; 0.79 = 0.88)	0.936 (0.88–0.99)	0.033	7,185 (OR = 1)	2,750 (OR = 0.97; 0.86–0.94)	0.984 (0.94–1.04)	0.53
Acute renal failure	5,665 (OR = 1)	4,810 (OR = 1.500; 1.44–1.56)	1.294 (1.24–1.35)	<0.001	15,410 (OR = 1)	8,855 (OR = 1.383; 1.35–1.42)	1.217 (1.18–1.26)	<0.001

## Patients Undergoing sAVR

Hospitalizations for sAVR dropped from 7.72 to 6.63/100,000 US adult in patients with diabetes (*p* = 0.025; Figure 2B). A similar trend was observed in non-diabetes patients in whom hospitalizations went down from 17.74 to 15.13/ 100 000 adult population, although statistical significance was not reached (*p* = 0.092). Mean age from 2012 to 2017 trended downwards (*p* = 0.001; Table 4). sAVR was increasingly performed in patients younger than 55 years; a converse trend was seen in patients aged between 75 and 84 years of age (*p* = 0.002) and >85 years (*p* < 0.001). Increasing proportions of patients identifying as Hispanic and Asians underwent sAVR (*p* = 0.002, *p* = 0.007, respectively). Contrary to patients with TAVR with diabetes, those hospitalized with sAVR did not show an increasing trend in hypertension, coronary artery disease, smoking, dyslipidemia, peripheral vascular disease, and renal failure. Age-adjusted mortality among patients with diabetes undergoing sAVR decreased from 3.4 to 2.4% (*p* < 0.001; Figure 3B). However, no significant changes were observed in non-diabetes mortality which decreased only from 3 to 2.8% (*p* = 0.09). There was no difference in the temporal trend of LoS, but total charges went up from $190,071 to $249,574 (*p* = 0.002; Figure 4B). In terms of post-surgical complications, upwards trends were seen in the need for permanent pacemakers (4.9–6.7%, *p* = 0.018), bleeding (2.5–3.4%, *p* = 0.032), acute renal failure (21.7–24.8%, *p* = 0.036), but not in stroke. A similar trend was observed in non-diabetes patients (Supplementary Table 2).

**Table 4:** Baseline characteristics of patients with diabetes undergoing sAVR.

	**2012**	**2013**	**2014**	**2015**	**2016**	**2017**	***P*** **-value**
**Age**							
Mean (SD)	70.40 (10.038)	70.26 (10.011)	69.51 (10.081)	69.48 (9.607)	68.45 (9.660)	68.01 (9.450)	0.001
<55	1,210 (6.7)	1,290 (7.5)	1,395 (8.4)	1,380 (7.7)	1,410 (8.3)	1,350 (8.7)	0.031
55–64	3,275 (18.1)	3,030 (17.7)	3,065 (18.5)	3,375 (18.8)	3,725 (22.0)	3,515 (22.6)	0.014
65–74	6,765 (37.3)	6,380 (37.2)	6,520 (39.4)	7,260 (40.5)	6,940 (41.0)	6,650 (42.8)	0.001
75–84	5,945 (32.8)	5,675 (33.1)	4,980 (30.1)	5,315 (29.7)	4,550 (26.9)	3,825 (24.6)	0.002
>84	940 (5.2)	780 (4.5)	600 (3.6)	580 (3.2)	320 (1.9)	195 (1.3)	<0.001
**Gender**							
Male	11,355 (62.6)	10,825 (63.1)	10,470 (63.2)	11,355 (63.4)	11,070 (65.3)	10,300 (66.3)	0.009
Female	6,780 (37.4)	6,330 (36.9)	6,090 (36.8)	6,555 (36.6)	5,875 (34.7)	5,235 (33.7)	0.009
**Race**							
White	13,975 (82.0)	13,295 (83.2)	12,885 (82.7)	13,945 (82.3)	12,860 (80.2)	11,795 (79.2)	0.055
Black	1,065 (6.2)	940 (5.9)	1,040 (6.7)	950 (5.6)	960 (6.0)	995 (6.7)	0.698
Hispanic	980 (5.7)	1,035 (6.5)	980 (6.3)	1,250 (7.4)	1,380 (8.6)	1,345 (9.0)	0.002
Asian	185 (1.1)	215 (1.3)	220 (1.4)	270 (1.6)	360 (2.2)	300 (2.0)	0.007
Native American	140 (0.8)	70 (0.4)	35 (0.2)	80 (0.5)	75 (0.5)	110 (0.7)	0.962
Other	705 (4.1)	425 (2.7)	425 (2.7)	445 (2.6)	390 (2.4)	340 (2.3)	0.049
**Income**							
Low	4,735 (26.6)	4,230 (25.2)	3,880 (23.9)	4,485 (25.4)	4,385 (26.3)	3,770 (24.6)	0.6
Low-Mid	4,725 (26.5)	4,685 (27.9)	4,730 (29.2)	4,400 (25.0)	4,515 (27.1)	4,300 (28.1)	0.923
High-Mid	4,495 (25.2)	4,445 (26.5)	4,050 (25.0)	4,495 (25.5)	420 (25.2)0	4,090 (26.7)	0.564
High	3,855 (21.6)	3,445 (20.5)	3,550 (21.9)	4,245 (24.1)	3,545 (21.3)	3,150 (20.6)	0.976
**Primary expected payer**							
Medicare	13,110 (72.3)	12,340 (72.0)	11,680 (70.6)	12,470 (69.7)	11,400 (67.3)	10,300 (66.4)	0.001
Medicaid	640 (3.5)	565 (3.3)	625 (3.8)	860 (4.8)	960 (5.7)	885 (5.7)	0.004
Private insurance	3,695 (20.4)	3,470 (20.2)	3,720 (22.5)	4,015 (22.4)	3,900 (23.0)	3,820 (24.6)	0.005
Self pay	305 (1.7)	245 (1.4)	245 (1.5)	250 (1.4)	175 (1.0)	190 (1.2)	0.037
No charge	20 (0.1)	45 (0.3)	25 (0.2)	15 (0.1)	10 (0.1)	20 (0.1)	0.374
Other	355 (2.0)	480 (2.8)	245 (1.5)	280 (1.6)	485 (2.9)	300 (1.9)	0.987
**Comorbidities**							
Obesity	5,490 (30.3)	5,675 (33.1)	5,670 (34.2)	6,425 (35.9)	6,505 (38.4)	6,085 (39.2)	<0.001
Hypertension	15,530 (85.6)	14,730 (85.9)	14,225 (85.9)	15,755 (88.0)	14,390 (84.9)	14,390 (84.9)	0.195
Smoking	5,440 (30.0)	5,270 (30.7)	6,255 (37.8)	6,250 (34.9)	5,445 (32.1)	5,040 (32.4)	0.64
Dyslipidemia	12,655 (69.8)	12,260 (71.5)	11,860 (71.6)	13,205 (73.7)	12,570 (74.2)	11,125 (71.6)	0.177
Elixhauser score, *n* (SD)	7.3749 (10.16033)	7.2957 (10.11426)	7.3822 (10.22661)	7.2236 (10.11324)	6.3910 (9.63573)	7.5231 (9.92958)	0.592
**Past medical history**							
Peripheral vascular disease	3,375 (18.6)	3,245 (18.9)	3,120 (18.8)	3,480 (19.4)	3,410 (29.1)	3,105 (20.0)	0.314
Renal failure	4,065 (22.4)	3,670 (21.4)	3,870 (23.4)	4,140 (23.1)	3,975 (23.5)	3,680 (23.7)	0.075
Coronary artery disease	10,825 (59.7)	11,020 (64.2)	10,360 (62.6)	11,145 (62.2)	10,755 (63.5)	9,985 (64.3)	0.172
**Hospital bed size**							
Small	226 (6.2)	233 (6.8)	299 (9.0)	290 (8.1)	301 (8.9)	310 (10.0)	0.013
Medium	664 (18.3)	634 (18.5)	806 (24.3)	907 (25.3)	773 (22.8)	732 (23.6)	0.108
Large	2,737 (75.5)	2,564 (74.7)	2,207 (66.6)	2,385 (66.6)	2,315 (68.3)	2,065 (66.5)	0.051
**Hospital location**							
Rural	113 (3.1)	109 (3.2)	66 (2.0)	68 (1.9)	62 (1.8)	75 (2.4)	0.14
Urban	3,514 (96.9)	3,322 (96.8)	3,246 (98.0)	3,514 (98.1)	3,327 (98.2)	3,032 (97.6)	0.14
**Hospital region**							
Northeast	904 (24.9)	802 (23.4)	753 (22.7)	816 (22.8)	702 (20.7)	609 (19.6)	0.002
Midwest	982 (27.1)	902 (26.3)	908 (27.4)	875 (24.4)	880 (26.0)	796 (25.6)	0.246
South	1,352 (37.3)	1,294 (37.7)	1,252 (37.8)	1,251 (34.9)	1,196 (35.3)	1,098 (35.3)	0.059
West	389 (10.7)	433 (12.6)	399 (12.0)	640 (17.9)	611 (18.0)	604 (19.4)	0.006
**Outcomes**							
Stroke	250 (1.4)	190 (1.1)	175 (1.1)	170 (0.9)	125 (0.7)	155 (1.0)	0.069
Pacemaker	895 (4.9)	965 (5.6)	820 (5.0)	1,035 (5.8)	1,040 (6.1)	1,045 (6.7)	0.018
Bleeding	450 (2.5)	345 (2.0)	410 (2.5)	460 (2.6)	560 (3.3)	525 (3.4)	0.032
Acute renal failure	3,940 (21.7)	3,565 (20.8)	3,705 (22.4)	4,360 (24.3)	3,895 (23.0)	3,860 (24.8)	0.036
Length of stay	11 (8)	10 (7)	10 (7)	10 (8)	10 (8)	10 (8)	0.621

### Comparison of Diabetes to Non-diabetes Patients

Contrary to TAVR, the mean (SD) age of patients with diabetes who underwent sAVR was higher than non-diabetes (Table 5). Of comorbidities compared, only PVD was more common among non-diabetes patients. Diabetes was associated with a higher adjusted mortality [OR = 1.115 (1.06–1.17), stroke [OR = 1.140 (1.05–1.23)], pacemaker requirement [OR = 1.089 (1.05–1.13)] and acute renal failure [OR = 1.217 (1.18–1.26)] (Table 3). Patients with diabetes had a marginally longer mean (SD) LoS [10(10) vs. 9(8) days, diabetes vs. non-diabetes, *p* < 0.001], and total charges [163,822 (116,172–236,122) vs. 158,210 (111,938–236,122) USD, diabetes vs. non-diabetes, *p* < 0.001].

**Table 5:** Comparison of patients with vs. without diabetes undergoing sAVR.

		**Diabetes**	**No diabetes**	***P*** **-value**
Age	Mean (SD)	69.59 (9.939)	66.93 (13.766)	<0.001
	<55	8,035 (7.9%)	41,345 (17.2%)	<0.001
	55–64	49,305 (20.5%)	19,985 (19.5%)	<0.001
	65–74	40,515 (39.6%)	72,650 (30.2%)	<0.001
	75–84	30,290 (29.6%)	64,475 (26.8%)	<0.001
	>84	3,415 (3.3%)	12,470 (5.2%)	<0.001
Gender	Male	65,375 (63.9%)	156,965 (65.3%)	<0.001
	Female	36,865 (36.1%)	83,280 (34.7%)	<0.001
Race	White	78,755 (81.6%)	190,485 (85.0%)	<0.001
	Black	5,950 (6.2%)	11,100 (5.0%)	<0.001
	Hispanic	6,970 (7.2%)	11,640 (5.2%)	<0.001
	Asian	1,550 (1.6%)	3,015 (1.3%)	<0.001
	Native American	510 (0.5%)	775 (0.3%)	<0.001
	Other	2,730 (2.8%)	7,050 (3.1%)	0.017
Income	Low	25,485 (25.4%)	51,770 (22.0%)	
	Low-Mid	27,355 (27.2%)	61,715 (26.2%)	<0.001
	High-Mid	25,775 (25.7%)	61,540 (26.1%)	<0.001
	High	21,790 (21.7%)	60,345 (25.6%)	<0.001
Primary expected payer	Medicare	71,300 (69.8%)	142,955 (59.6%)	<0.001
	Medicaid	4,535 (4.4%)	14,025 (5.8%)	<0.001
	Private insurance	22,620 (22.1%)	72,290 (30.1%)	<0.001
	Self pay	1,410 (1.4%)	4,990 (2.1%)	<0.001
	No charge	135 (0.1%)	635 (0.3%)	<0.001
	Other	2,145 (2.1%)	4,980 (2.1%)	<0.001
Obesity		35,850 (35.1%)	40,370 (16.8%)	<0.001
Hypertension		85,175 (83.3%)	164,160 (68.3%)	<0.001
Smoking		33,700 (33.0%)	78,910 (32.8%)	0.508
Dyslipidemia		73,675 (72.1%)	129,950 (54.1%)	<0.001
Peripheral vascular disease		19,735 (19.3%)	56,770 (23.6%)	<0.001
Renal failure		23,400 (22.9%)	29,930 (12.5%)	<0.001
Coronary artery disease		64,090 (62.7%)	115,100 (47.9%)	<0.001
Elixhauser score	Mean (SD)	8.4487 (9.89012)	7.1612 (10.00983)	<0.001
Hospital bedsize	Small	8,295 (8.1%)	19,320 (8.0%)	<0.001
	Medium	22,580 (22.1%)	50,010 (20.8%)	<0.001
	Large	71,365 (69.8%)	170,915 (71.1%)	0.29
Hospital location	Rural	2,465 (2.4%)	5,060 (2.1%)	0.01
	Urban	99,775 (97.6%)	235,185 (97.9%)	0.01
Hospital region	Northeast	22,930 (22.4%)	56,565 (23.5%)	<0.001
	Midwest	26,715 (26.1%)	62,030 (25.8%)	<0.001
	South	37,215 (36.4%)	82,520 (34.3%)	<0.001
	West	15,380 (15.0%)	39,130 (16.3%)	0.012

## Predictors of Mortality in Both Interventions

Analysis of age in patients with diabetes hospitalized with TAVR revealed a lower odds of mortality in all age categories above 55 compared to patients younger than 55 years old (Table 6). Interestingly, blacks had a 34% less risk of dying than white Americans whereas the risk of native Americans increased by almost 4-fold. No effect of gender was observed in all patients. Peripheral vascular disease, coronary artery disease, renal failure, and the Elixhauser score increased the risk of death but paradoxically, obesity, smoking and dyslipidemia lowered it. However, predictors of mortality in patients with diabetes hospitalized for sAVR were different. Patients older than 84 years of age have a higher risk of mortality, so did females and patients who belong to ethnic minorities. Coronary artery disease, peripheral vascular disease, and renal failure increased the risk of death, while hypertension, smoking, and dyslipidemia decreased it.

**Table 6:** Predictors of mortality in patients with diabetes undergoing SAVR and TAVR.

		**TAVR**			**sAVR**		
		**OR**	**95% CI**	***P*** **-value**	**OR**	**95% CI**	***P*** **-value**
Age	<55	Ref	Ref	–	Ref	Ref	–
	55–64	0.442	0.28–0.71	0.001	0.79	0.75–1.02	0.093
	65–74	0.291	0.19–0.45	<0.001	0.92	0.81–1.05	0.102
	75–84	0.24	0.16–0.37	<0.001	1.167	1.02–1.33	<0.001
	>84	0.38	0.25–0.58	<0.001	1.55	1.29–1.86	0.021
Gender	Male	Ref	Ref	–	Ref	Ref	–
	Female	0.977	0.86–1.11	0.11	1.572	1.47–1.68	<0.001
Race	White	Ref	Ref	–	Ref	Ref	–
	Black	0.657	0.46–0.95	0.025	1.191	1.04–1.36	<0.001
	Hispanic	1.196	0.91–1.58	0.206	1.338	1.12–1.51	<0.001
	Asian	1.491	0.95–2.34	0.081	1.581	1.26–1.99	<0.001
	Native American	3.977	2.09–7.58	<0.001	1.89	1.30–2.75	<0.001
	Other	0.954	0.64–1.43	0.82	1.378	1.15–1.65	<0.001
Income	Low	Ref	Ref	–	Ref	Ref	–
	Low-Mid	0.721	0.90–0.87	0.001	0.975	0.89–1.07	<0.001
	High-Mid	0.992	0.83–1.19	0.929	0.873	0.80–0.96	<0.001
	High	0.969	0.81–1.16	0.73	0.707	0.64–0.78	<0.001
Primary expected payer	Medicare	Ref	Ref	–	Ref	Ref	–
	Medicaid	1.578	1.01–2.47	0.047	0.997	0.85–1.17	<0.001
	Private insurance	0.659	0.49–0.89	0.007	0.543	0.48–0.60	<0.001
	Self pay	1.107	0.46–2.69	0.822	0.979	0.75–1.28	0.62
	No charge	1.384	0.42–2.56	0.999	0.812	0.33–1.98	0.888
	Other	0.295	0.12–0.71	0.007	1.084	0.87–1.35	<0.001
Obesity	No	Ref	Ref	–	Ref	Ref	–
	Yes	0.81	0.69–0.95	0.008	0.977	0.91–1.05	0.10
Hypertension	No	Ref	Ref	–	Ref	Ref	–
	Yes	1.092	0.96–1.25	0.19	0.845	0.79–0.91	<0.001
Smoking	No	Ref	Ref	–	Ref	Ref	–
	Yes	0.565	0.48–0.66	<0.001	0.606	0.56–0.66	<0.001
Dyslipidemia	No	Ref	Ref	Ref	Ref	Ref	
	Yes	0.598	0.52–0.68	<0.001	0.588	0.55–0.63	<0.001
Peripheral vascular disease	No	Ref	Ref	–	Ref	Ref	–
	Yes	1.25	1.09–1.44	0.002	1.447	1.34–1.56	<0.001
Renal Failure	No	Ref	Ref	–	Ref	Ref	–
	Yes	1.52	1.34–1.73	<0.001	2.172	2.03–2.33	<0.001
Coronary artery disease	No	Ref	Ref	–	Ref	Ref	–
	Yes	1.783	1.56–2.03	<0.001	1.24	1.16–1.33	<0.001
Elixhauser score		1.084	1.08–1.09	<0.001	1.068	1.07–1.07	<0.001
Hospital bedsize	Small	Ref	Ref	–	Ref	0.659	–
	Medium	0.919	0.67–1.27	0.607	0.93	0.82–1.08	0.18
	Large	0.925	1.01–0.76	0.925	0.953	0.84–1.08	0.039
Hospital location	Rural	Ref	Ref	–	Ref	Ref	–
	Urban	0.793	0.42–1.49	0.471	0.72	0.60–0.87	0.463
Hospital region	Northeast	Ref	Ref	–	Ref	<0.001	–
	Midwest	1.419	1.16–1.73	0.001	1.587	1.43–1.76	<0.001
	South	1.641	1.37–1.97	<0.001	1.73	1.57–1.91	<0.001
	West	0.952	0.75–1.21	0.688	1.592	1.42–1.79	<0.001

## Discussion

When it was first introduced in 2002, TAVR was initially reserved as a treatment for patients with severe AS but whose surgical risk was prohibitive for undergoing sAVR (14). Further, several studies have compared TAVR to the conventional sAVR. Initially, TAVR was non-inferior and superior in Partner 1A (15) and CoreValve Extreme Risk Trials (16). Subsequently, studies proved the superiority of TAVR in high-risk patients (17), non-inferior in intermediate-risk (18), and superior in low-risk patients (19). We recently showed that most post-procedural aortic valve function parameters assessed by echocardiography also favor TAVR (20). With newer studies proving not only safety but also the superiority of TAVR as an alternative to sAVR among patients with diverse surgical risk profiles (17–19), the proportion of TAVR procedures relative to sAVR procedures conducted annually is on the rise, as seen in our study in both diabetes and non-diabetes patients.

The comparatively minimally invasive nature of TAVR relative to sAVR makes it an attractive option for several candidates. The increasing proportion of patients between 55 and 74 of age undergoing TAVR is a testament to the growing popularity of TAVR. Despite the increasing proportions of people with diabetes with comorbidities such as obesity, smoking, and a higher Elixhauser score undergoing TAVR, mortality trend analysis shows a significant decline in mortality. This is likely due to increased provider experience, valve technology developments, and delivery system optimization, as reported in a recent meta-analysis (21). Further evidencing positive trends showing improved outcomes through experience are declining trends of post-procedural stroke and acute renal failure, all of which are encouraging regarding the future of TAVR among the diabetes population.

In our study, diabetes was not associated with an increased in-hospital mortality risk in TAVR patients, which is aligned with previous findings from the Sheba Medical Center database (22) and recent findings from the Society of Thoracic Surgeons/American College of Cardiology Transcatheter Valve Therapy Registry (23). Nevertheless, diabetes is associated with an increased risk of renal failure, pacemaker requirement, and stroke in TAVR, previously reported in other studies. In a recent analysis of 3 Swedish national registries, diabetes increased the risk of stroke by almost 60% (24), resulting from hyper aggregation often encountered in this pathology, leading to an increased risk of cerebral embolization. A meta-analysis of 64 studies that included over 38 000 TAVR patients reported an increase of 30% in acute renal failure (25); patients with diabetes often suffer from diabetic nephropathy and have a lower age-adjusted estimated glomerular filtration rate (26). The increased need for pacemaker requirement in patients with diabetes is unknown; it might be due to the higher prevalence of complete heart block in diabetes patients (27, 28).

Head-to-head randomized controlled trials comparing TAVR to sAVR in patients with diabetes are missing. A *post-hoc* analysis of the PARTNER study, a randomized controlled trial of TAVR vs. sAVR in high-risk patients with severe AS, TAVR reduced 1-year mortality in patients with diabetes compared to sAVR (29). Interestingly, no survival benefit was noted in non-diabetes patients. In a recent case-control study that assessed both interventions in patients with diabetes, Khan et al. reported lower mortality but higher post-procedural complications in patients who underwent TAVR (30). However, patients were initially assigned to either one of both interventions according to their surgical risk and not diabetes, which is the significant bias of this retrospective analysis. In recent years, TAVR has been praised for its durability. Blackman et al. reported only <1% of severe valve degeneration in the U.K. TAVI registry, 5–10 years post-procedure (31). The center for heart valve innovation (Canada) recently reported that the rate of structural valve deterioration/bioprosthetic valve failure at four, six, eight, and 10 years was 0.4, 1.7, 4.7, and 6.55, respectively (32). Although both studies included patients with diabetes, the association between valvular deterioration and diabetes was not assessed separately. However, we believe that TAVR is a safe short and long-term procedure for aortic valve replacement in relatively young patients with diabetes.

Among predictors of mortality, it is interesting to note that smoking and dyslipidemia appear to confer a protective effect against mortality, which has previously been documented in other analyses of diabetes patients hospitalized for MI (33), heart failure (34), or stroke in the NIS database (35). This described “smoking paradox” has an unclear etiology, as smoking is an established risk factor in developing vascular disease (36). It has been proposed that this effect may be due to the differential impact of antiplatelet agents on smokers relative to non-smokers (37), or perhaps the younger age of presentation of smokers relative to non-smokers for cardiac interventions (38).

Our study has several limitations. Firstly, it relies on an administrative data input primarily for reimbursement purposes; the NIS was not built as a medical cohort *per se* and is therefore subject to coding errors. Secondly, the NIS is a retrospective observational database. Although it serves the purpose of trend analysis, only association with cardiovascular outcomes may be made from our results, while no causative relations may be inferred. Lastly, our data may have been more meaningful had we been able to include in our multivariable analysis several co-founding factors such as medications and other significant predictors of mortality in patients hospitalized for AVR such as echocardiographic parameters, surgical risk scores, and diabetes duration, severity, and control for those patients with diabetes. Despite those limitations, we believe that our study could elucidate the recent trend and outcome of diabetes hospitalized for aortic valve replacement.

## Conclusion

The recent temporal trend shows that hospitalizations for TAVR in patients with diabetes are on the rise, whereas that of sAVR are decreasing. In both aortic valvular replacement procedures, mortality is decreasing in those patients. However, diabetes is still associated with increased risk of stroke, acute renal failure, and pacemaker requirement in both techniques. Further, diabetes is still associated with an increased risk of mortality in sAVR but not in TAVR.

## Data Availability Statement

The raw data supporting the conclusions of this article will be made available by the authors, upon reasonable request.

## Author Contributions

CAK: conception and design. SD: statistical analysis. SK, JA, HJ, and CAK: analysis and interpretation of the data. SK: drafting the first manuscript. All authors revised the final version of the final manuscript and approved it. All authors contributed to the article and approved the submitted version.

## Conflict of Interest

The authors declare that the research was conducted in the absence of any commercial or financial relationships that could be construed as a potential conflict of interest.

## Publisher's Note

All claims expressed in this article are solely those of the authors and do not necessarily represent those of their affiliated organizations, or those of the publisher, the editors and the reviewers. Any product that may be evaluated in this article, or claim that may be made by its manufacturer, is not guaranteed or endorsed by the publisher.
