# Supplementary material for: Trends and Outcomes of Aortic Valve Replacement in Patients With Diabetes in the US
Source: Front Cardiovasc Med. 2022 Mar 18;9:844068. doi: 10.3389/fcvm.2022.844068 (PMC8971926; doi:10.3389/fcvm.2022.844068)
Supplement: Supplementary file 1 [file Data_Sheet_1.docx]

**Supplementary table 1.** Baseline characteristics of non-diabetic patients undergoing TAVR.

|  | **2012** | **2013** | **2014** | **2015** | **2016** | **2017** | **P value** |
| --- | --- | --- | --- | --- | --- | --- | --- |
| **Age** |  | | | | | | |
| Mean (SD) | 81.77 (8.801) | 82.33 (7.898) | 82.17 (8.166) | 81.90 (8.444) | 81.45 (8.099) | 80.75 (8.495) | 0.085 |
| <55 | 95 (2.1) | 80 (1.0) | 140 (1.2) | 260 (1.6) | 250 (1.0) | 375 (1.2) | 0.229 |
| 55-64 | 160 (3.6) | 240 (3.1) | 330 (2.9) | 490 (2.9) | 695 (2.8) | 1130 (3.7) | 0.917 |
| 65 - 74 | 430 (9.7) | 765 (9.7) | 1100 (9.8) | 1730 (10.3) | 3255 (13.3) | 4680 (15.1) | 0.019 |
| 75-84 | 1605 (36.1) | 2890 (36.8) | 4155 (36.9) | 6230 (37.2) | 9715 (39.6) | 12335 (39.9) | 0.007 |
| >84 | 2150 (48.4) | 3880 (49.4) | 5530 (49.1) | 8000 (47.8) | 10615 (43.3) | 12405 (40.1) | 0.027 |
| **Gender** |  | | | | | | |
| Male | 2240 (50.5) | 3750 (47.7) | 5995 (53.3) | 8415 (50.4) | 12930 (52.7) | 16055 (51.9) | 0.307 |
| Female | 2200 (49.5) | 4105 (52.3) | 5260 (46.7) | 8295 (49.6( | 11600 (47.3) | 14877 (48.1) | 0.307 |
| **Race** |  |  |  |  |  |  |  |
| White | 3625 (86.8) | 6545 (90.6) | 9470 (91.5) | 13855 (88.8) | 20645 (88.5) | 26645 (89.4) | 0.807 |
| Black | 155 (3.7) | 210 (2.9) | 320 (3.1) | 590 (3.8) | 875 (3.8) | 960 (3.2) | 0.819 |
| Hispanic | 95 (2.3) | 170 (2.4) | 225 (2.2) | 630 (4.0) | 830 (3.6) | 1165 (3.9) | 0.036 |
| Asian | 30 (0.7) | 35 (0.5) | 70 (0.7) | 140 (0.9) | 260 (1.1) | 320 (1.1) | 0.02 |
| Native American | 15 (0.4) | 10 (0.1) | 10 (0.1) | 15 (0.1) | 60 (0.3) | 75 (0.3) | 0.94 |
| Other | 255 (6.1) | 255 (3.5) | 260 (2.5) | 375 (2.4) | 660 (2.8) | 635 (2.1) | 0.056 |
| **Income** |  | | | | | | |
| Low | 950 (21.6) | 1600 (20.8) | 2160 (19.4) | 3290 (20.0) | 5045 (20.9) | 6000 (19.7) | 0.259 |
| Low-Mid | 925 (21.1) | 1855 (24.2) | 3100 (27.9) | 4125 (25.1) | 5785 (24/0) | 7725 (25.4) | 0.387 |
| High-Mid | 1165 (26.5) | 1965 (25.6) | 2850 (25.6) | 4380 (26.7) | 6630 (27.5) | 8285 (27.2) | 0.126 |
| High | 1350 (30.8) | 2255 (29.4) | 3005 (27.0) | 4630 (28.2) | 6655 (27.6) | 8440 (27.7) | 0.086 |
| **Primary Expected Payer** |  | | | | | | |
| Medicare | 3980 (89.6) | 7085 (90.3) | 10260 (91.3) | 15120 (90.6) | 22435 (91.6) | 27915 (90.5) | 0.234 |
| Medicaid | 30 (0.7) | 60 (0.8) | 90 (0.8) | 150 (0.9) | 225 (0.9) | 360 (1.2) | 0.015 |
| Private insurance | 360 (8.1) | 540 (6.9) | 720 (6.4) | 1155 (6.9) | 1435 (5.9) | 2110 (6.8) | 0.156 |
| Self Pay | 25 (0.6) | 30 (0.4) | 60 (0.5) | 110 (0.7) | 150 (0.6) | 70 (0.2) | 0.485 |
| No charge | 5 (0.1) | 0 (0.0) | 0 (0.0) | 5 (0.0) | 10 (0.0) | 5 (0.0) | 0.158 |
| Other | 40 (0.9) | 130 (1.7) | 105 (0.9) | 150 (0.9) | 245 (1.0) | 390 (1.3) | 0.975 |
| **Comorbidities** |  |  |  |  |  |  |  |
| Obesity | 380 (8.6%) | 765 (9.7%) | 1180 (10.5%) | 1905 (11.4%) | 2595 (10.6%) | 4265 (13.8%) | 0.028 |
| Hypertension | 3515 (79.2%) | 6070 (77.3%) | 9130 (81.1%) | 13895 (83.2%) | 19185 (78.2%) | 19185 (78.2%) | 0.223 |
| Smoking | 1100 (24.8) | 2000 (25.5) | 3495 (31.1) | 5425 (32.5) | 8085 (33.0) | 10560 (34.1) | 0.006 |
| Dyslipidemia | 2575 (58.0) | 4630 (58.9) | 7145 (63.5) | 10820 (64.8) | 15625 (63.7) | 19565 (63.3) | 0.059 |
| Elixhauser score, n (SD) | 8.7971 (9.58960) | 8.7941 (9.71891) | 10.8054 (10.47316) | 7.4229 (9.00827) | 10.6523 (8.19167) | 10.8636 (7.93094) | 0.426 |
| **Past Medical History** |  |  |  |  |  |  |  |
| Peripheral vascular disease | 1425 (32.1) | 2210 (28.1) | 3265 (29.0) | 4655 (27.9) | 6260 (25.5) | 7895 (25.5) | 0.012 |
| Renal failure | 1370 (30.9) | 2440 (31.1) | 3765 (33.5) | 5005 (30.0) | 7320 (29.8) | 8535 (27.6) | 0.153 |
| Coronary artery disease | 2810 (63.3) | 5095 (64.9) | 7635 (67.8) | 11405 (68.3) | 17170 (70.0) | 21070 (68.1) | 0.03 |
| **Hospital bed size** |  |  |  |  |  |  |  |
| Small | 28 (3.2) | 49 (3.1) | 117 (5.2) | 177 (5.3) | 298 (6.1) | 408 (6.6) | 0.003 |
| Medium | 112 (12.6) | 233 (14.8) | 398 (17.7) | 683 (20.4) | 881 (18.0) | 1203 (19.5) | 0.032 |
| Large | 748 (84.2) | 1289 (82.0) | 1736 (77.1) | 2482 (74.3) | 3727 (76.0) | 4574 (74.0) | 0.012 |
| **Hospital location** |  |  |  |  |  |  |  |
| Rural | 3 (0.3) | 19 (1.2) | 17 (0.8) | 33 (1.0) | 36 (0.7) | 62 (1.0) | 0.348 |
| Urban | 885 (99.7) | 1552 (98.8) | 2234 (99.2) | 3309 (99.0) | 4870 (99.3) | 6123 (99.0) | 0.465 |
| **Hospital Region** |  |  |  |  |  |  |  |
| Northeast | 232 (26.1) | 476 (30.3) | 626 (27.8) | 860 (25.7) | 1273 (25.9) | 1507 (24.4) | 0.187 |
| Midwest | 199 (22.4) | 345 (22.0) | 550 (24.4) | 762 (22.8) | 1079 (22.0) | 1380 (22.3) | 0.815 |
| South | 359 (40.4) | 591 (37.6) | 826 (36.7) | 1083 (32.4) | 1611 (32.8) | 2047 (33.1) | 0.012 |
| West | 98 (11.0) | 159 (10.1) | 249 (11.1) | 637 (19.1) | 943 (19.2) | 1251 (20.2) | 0.015 |
| **Outcomes** |  |  |  |  |  |  |  |
| Stroke | 60 (1.4) | 110 (1.4) | 135 (1.2) | 185 (1.1) | 150 (0.6) | 235 (0.8) | 0.013 |
| Pacemaker | 340 (7.7) | 720 (9.2) | 1155 (10.3) | 1865 (11.2) | 2500 (10.2) | 3070 (9.9) | 0.148 |
| Bleeding | 215 (4.8) | 350 (4.5) | 535 (4.8) | 625 (3.7) | 1275 (5.2) | 1090 (3.5) | 0.387 |
| Acute renal failure | 670 (15.1) | 1460 (18.6) | 1900 (16.9) | 2045 (12.2) | 2460 (10.0) | 2745 (8.9) | 0.033 |
| **Length of stay** | 8 (6) | 7 (7) | 7 (6) | 5 (6) | 4 (5) | 4 (4) | <0.001 |

**Supplementary Table 2**. Baseline characteristics of non-diabetic patients undergoing SAVR.

|  | **2012** | **2013** | **2014** | **2015** | **2016** | **2017** | **P value** |
| --- | --- | --- | --- | --- | --- | --- | --- |
| **Age** |  |  |  |  |  |  |  |
| Mean (SD) | 67.92 (14.006) | 67.52 (13.857) | 67.03 (13.731) | 66.46 (13.617) | 65.48 (13.644) | 64.96 (13.014) | <0.001 |
| <55 | 6790 (16.3) | 6270 (16.2) | 6675 (16.7) | 7365 (17.6) | 7485 (18.4) | 6760 (18.1) | 0.007 |
| 55-64 | 7555 (18.1) | 7440 (19.2) | 8065 (20.1) | 8705 (20.8) | 8805 (21.6) | 8735 (23.4) | <0.001 |
| 65 - 74 | 11455 (27.5) | 11055 (28.6) | 11610 (29.0) | 12635 (30.2) | 13165 (32.3) | 12730 (34.2) | 0.001 |
| 75-84 | 12690 (30.5) | 11250 (29.1) | 11395 (28.5) | 10995 (26.3) | 9950 (24.4) | 8195 (22.0) | <0.001 |
| >84 | 3150 (7.6) | 2705 (5.7) | 2285 (5.7) | 2160 (5.2) | 1320 (3.2) | 850 (2.3) | 0.158 |
| **Gender** |  |  |  |  |  |  |  |
| Male | 26545 (63.7) | 24840 (64.2) | 26075 (65.1) | 27435 (65.5) | 27020 (66.3) | 25050 (67.2) | <0.001 |
| Female | 15095 (36.3) | 13880 (35.8) | 13955 (34.9) | 14425 (34.5) | 13705 (33.7) | 12220 (32.8) | <0.001 |
| **Race** |  | | | | | | |
| White | 32605 (84.4) | 30640 (86.0) | 31885 (86.4) | 33020 (84.9) | 32550 (84.6) | 29785 (83.8) | 0.349 |
| Black | 1930 (5.0) | 1725 (4.8) | 1785 (4.8) | 2045 (5.3) | 1860 (4.8) | 1755 (4.9) | 0.11 |
| Hispanic | 1530 (4.0) | 1530 (4.3) | 1800 (4.9) | 2160 (5.6) | 2335 (6.1) | 2285 (6.4) | <0.001 |
| Asian | 410 (1.1) | 460 (1.3) | 435 (1.2) | 525 (1.4) | 615 (1.6) | 570 (1.6) | 0.007 |
| Native American | 225 (0.6) | 110 (0.3) | 65 (0.2) | 145 (0.4) | 85 (0.2) | 145 (0.4) | 0.448 |
| Other | 1930 (5.0) | 1160 (3.3) | 945 (2.6) | 990 (2.5) | 1015 (2.6) | 1010 (2.8) | 0.095 |
| **Income** |  | | | | | | |
| Low | 9705 (23.8) | 8370 (22.2) | 8020 (20.5) | 8895 (21.6) | 8885 (22.3) | 7895 (21.6) | 0.346 |
| Low-Mid | 10555 (25.9) | 9870 (26.1) | 10965 (28.0) | 10385 (25.2) | 10300 (25.8) | 9640 (26.4) | 0.899 |
| High-Mid | 10040 (24.6) | 9830 (26.0) | 10220 (26.1) | 10880 (26.4) | 10850 (27.2) | 9720 (26.6) | 0.031 |
| High | 10460 (25.7) | 9715 (25.7) | 9995 (25.5) | 10990 (26.7) | 9895 (24.8) | 929 (25.4) | 0.62 |
| **Primary Expected Payer** |  | | | | | | |
| Medicare | 26000 (62.5) | 23885 (61.7) | 24100 (60.4) | 24685 (59.0) | 23395 (57.5) | 20890 (56.1) | <0.001 |
| Medicaid | 1790 (4.3) | 1680 (4.3) | 2440 (6.1) | 2635 (6.3) | 2910 (7.2) | 2570 (6.9) | 0.008 |
| Private insurance | 11750 (28.3) | 11120 (28.7) | 11860 (29.7) | 12830 (30.7) | 12610 (31.0) | 12120 (32.6) | <0.001 |
| Self Pay | 1025 (2.5) | 955 (2.5) | 765 (1.9) | 700 (1.7) | 760 (1.9) | 785 (2.1) | 0.172 |
| No charge | 150 (0.4) | 170 (0.4) | 105 (0.3) | 60 (0.1) | 100 (0.2) | 50 (0.1) | 0.017 |
| Other | 855 (2.1) | 875 (2.3) | 655 (1.6) | 895 (2.1) | 900 (2.2) | 800 (2.1) | 0.934 |
| **Comorbidities** |  |  |  |  |  |  |  |
| Obesity | 5575 (13.4) | 5755 (14.9) | 6800 (17.0) | 7480 (17.9) | 7665 (18.8) | 7095 (19.0) | 0.002 |
| Hypertension | 29120 (69.9) | 27165 (70.2) | 28460 (71.1) | 29885 (71.4) | 28640 (70.3) | 28640 (70.3) | 0.196 |
| Smoking | 13330 (32.0) | 13265 (34.3) | 14590 (36.4) | 14865 (35.5) | 11570 (28.4) | 11290 (30.3) | 0.354 |
| Dyslipidemia | 22185 (53.3) | 21420 (55.3) | 22150 (55.3) | 22950 (54.8) | 21955 (53.9) | 19290 (51.8) | 0.338 |
| Elixhauser score, n (SD) | 8.3182 (10.09012) | 8.5002 (10.01083) | 8.7482 (10.14455) | 8.7863 (10.38079) | 8.0143 (9.34334) | 9.0289 (9.50009) | 0.547 |
| **Past Medical History** |  | | | | | | |
| Peripheral vascular disease | 8560 (20.6) | 8105 (20.9) | 8720 (21.8) | 9825 (23.5) | 10910 (26.8) | 10650 (28.6) | 0.002 |
| Renal failure | 5245 (12.6) | 4740 (12.2) | 5370 (13.4) | 5265 (12.6) | 4980 (12.2) | 4330 (11.6) | 0.293 |
| Coronary artery disease | 19560 (47.0) | 18290 (47.2) | 19175 (47.9) | 19790 (47.3) | 20215 (49.6) | 18070 (48.5) | 0.079 |
| **Hospital bed size** |  | | | | | | |
| Small | 576 (6.9) | 520 (6.7) | 682 (8.5) | 699 (8.3) | 716 (8.8) | 671 (9.0) | 0.015 |
| Medium | 1493 (17.9) | 1363 (17.6) | 1856 (23.2) | 1928 (23.0) | 1688 (20.7) | 1674 (22.5) | 0.659 |
| Large | 6259 (75.2) | 5861 (75.7) | 5468 (68.3) | 5745 (68.6) | 5741 (70.5) | 5109 (68.5) | 0.081 |
| **Hospital location** |  | | | | | | |
| Rural | 232 (2.8) | 210 (2.7) | 151 (1.9) | 143 (1.7) | 148 (1.8) | 128 (1.7) | 0.021 |
| Urban | 8096 (97.2) | 7534 (97.3) | 7855 (98.1) | 8229 (98.3) | 7997 (98.2) | 7326 (98.3) | 0.021 |
| **Hospital Region** |  | | | | | | |
| Northeast | 2115 (25.4) | 1885 (24.3) | 1975 (24.7) | 1911 (22.8) | 1801 (22.1) | 1626 (21.8) | 0.003 |
| Midwest | 2139 (25.7) | 2032 (26.2) | 2172 (27.1) | 2096 (25.0) | 2070 (25.4) | 1897 (25.4) | 0.34 |
| South | 3024 (36.3) | 2778 (35.9) | 2814 (35.1) | 2775 (33.1) | 2695 (33.1) | 2418 (32.4) | 0.002 |
| West | 1050 (12.6) | 1049 (13.5) | 1045 (13.1) | 1590 (19.0) | 1579 (19.4) | 1513 (20.3) | 0.009 |
| **Outcomes** |  | | | | | | |
| Stroke | 555 (1.3) | 535 (1.4) | 450 (1.1) | 365 (0.9) | 440 (1.1) | 365 (1.0) | 0.088 |
| Pacemaker | 1955 (4.7) | 2010 (5.2) | 2015 (5.0) | 2200 (5.3) | 2385 (5.9) | 2275 (6.1) | 0.006 |
| Bleeding | 1025 (2.5) | 885 (2.3) | 1185 (3.0) | 1135 (2.7) | 1430 (3.5) | 1525 (4.1) | 0.017 |
| Acute renal Failure | 6485 (15.6) | 6205 (16.0) | 6805 (17.0) | 7045 (16.8) | 6995 (17.2) | 6640 (17.8) | 0.003 |
| **Length of stay** | 10 (9) | 10 (9) | 10 (9) | 9 (9) | 9 (9) | 9 (8) | 0.914 |
